# Use of ultrasound to enhance PEI-mediated gene delivery *in vivo* on diabetic model

**DOI:** 10.1186/1878-5085-5-S1-A76

**Published:** 2014-02-11

**Authors:** Rostyslav V Bubnov, Olena K Toporova, Dmytro M Irodov, Tamara P Gulko, Tetiana P Ruban, Pavlo V Morgunov, Vitaliy A Kordium

**Affiliations:** 1 Clinical hospital “Pheophania” of State Affairs Department, Kyiv, Ukraine; 2 Institute of Molecular Biology and Genetics of NAS of Ukraine, Kyiv, Ukraine

## Background and aims

Gene therapy has strong therapeutic potential for the personalized treatment of type 1 diabetes. Local gene delivery methods for gene therapy are a promising strategy to increase the therapeutic genes concentration at the target location. Transient ultrasound can cause the local increase of cell membrane permeability thus promoting the penetration of transfection particles into the cell.

## The objective

of this work is to evaluate transfection efficiency and safety for intrahepatic polyplex-mediated gene delivery by sonoporation.

## Methods

Polyplexes of expression vector DNAs complexed to galactose-bearing polyethylenimine (L-PEI) have been used. Expression vectors are two plasmids containing expression cassette for full-size human preproinsulin gene controlled with CMV promoter and flanked by inverted terminal repeats of AAV (pTRhins) and the same cassette for marker gfp gene (pTRegfp). Animals were injected under US guidance with polyplexes in a dose of 40 µg pTRegfp /0.7 ml (rat) and of 15 µg pTRhins /0.15 ml (diabetic mouse) into the liver parenchyma of subdiaphragmal segments using 31 G needles. Afterwards injection locus in depth of 1 cm during 180 sec was insonated by 130 Db ultrasound using multifrequency 3-5 MHz probe.

## Results

Sonoporation is able to increase polyplex gene transfer to liver cells *in vivo*. Flow cytometry analysis of primary hepatocytes isolated from the liver of experimental rats showed that ultrasound -enhanced polyplex gene transfer was highly localized, and was superior to all controls. At least 42% of the liver cells *in vivo* can be transfected in this way with ultrasound exposure versus 1.2% without it. Hypoglycemic effect of the insulin gene delivery followed by 3 min US exposure was observed on the third day: glucose level of diabetic mice (hyperglycemic 6- week) decreased in average by 30%. Nevertheless sonoporation does not increase naked plasmid DNA (without L-PEI ) transfection efficiency. A week and a month after the procedure serial sections from rat liver injected with polyplexes containing 40 µg marker plasmid or saline solution alone exposed to US and without it was analyzed for the presence of inflammation. Pathomorphological and histological analysis of experimental livers revealed no inflammatory processes in tissues, and any detectable side effects of US-enhanced gene delivery were seen. Experimental mice and rat liver DNAs were positive in transgene PCR for 3 months after gene delivery.

## Conclusions

Our results demonstrate that polyplex gene transfer by US exposure is effective, robust and feasible and illustrate the potential of ultrasound-triggered gene delivery technology for gene therapy.

## Outlook and expert recommendations

As a follow-up to this study, it is recommended to create project to study and implement diabetic gene therapy with drug delivery systems for personalized treatment of type 1 diabetes.
